# Numerical Simulation and Experimental Analysis on Seam Feature Size and Deformation for T-Joint Laser–GMAW Hybrid Welding

**DOI:** 10.3390/ma17010228

**Published:** 2023-12-31

**Authors:** Nai-Kun Wei, Jin Shi, Run-Dang Yang, Jun-Tong Xi, Xiao-Meng Luo, Xu-Yue Yin, Rui-Xue Zhang

**Affiliations:** 1 School of Mechanical Engineering, Shanghai Jiao Tong University, Shanghai 200240, China; 2 Shanghai Shipbuilding Technology Research Institute, Shanghai 200032, China; 3 National Engineering Research Center of Ship Intelligent Manufacturing, Shanghai 200032, China

**Keywords:** laser–GMAW hybrid welding, thermal dynamic simulation, seam feature size, deformation control, numerical simulation

## Abstract

As an innovative technique, laser–GMAW hybrid welding manifests significant superiority in enhancing welding productivity and quality, albeit the optimization of process parameters poses a challenge for practical application. The present manuscript elucidates the influence of process parameters on the dimensional characteristics of the welding seam and the distortion of 8 mm T-joints in the context of laser–GMAW hybrid welding, and channels both simulation and experimentation. The outcomes denote that the dual conical model serves as an efficacious aid for the numerical simulation of T-joint laser–GMAW hybrid welding. Furthermore, the repercussions of process parameters on welding seam dimensional characteristics remain consistently similar in both the simulation and experimental results. From the simulation outcomes, it becomes apparent that the distortion of the base material can be efficiently managed by implementing anti-distortion measures. This inquiry offers both a theoretical and experimental foundation for optimizing process parameters of T-joint laser–GMAW hybrid welding, presenting certain engineering applicability.

## 1. Introduction

Maritime slim panels are prevalently utilized in the deck constructs, wall veneers, platforms, and superstructural elements of luxury cruise ships and surface seafaring vessels. A pair of considerably advanced welding techniques, namely subaqueous arc welding and semi-automatic gas shielded welding, are extensively employed in domestically renowned shipyards to execute the fabrication of these maritime slim panels. Nonetheless, the utilization of such conventional welding methodologies has culminated in substantial distortions in the welding process and low efficacy with augmented intricacy in maintaining control over the quality of welding. These factors no longer satiate the requisite precision and cycle of voluminous slim panel manufacturing. Conversely, the application of laser composite welding posits the benefits of swift speed, amplified efficiency, enhanced flexibility, diminished heat input, potent penetration capacity, and the weld depth to breadth ratio can attain a proportion exceeding 10:1 [1,2].

In this sophisticated welding technique, a pair of heat sources work in tandem to regulate the thermal input within the weld zone. One potential heat source might be the gas metal arc welding (GMAW) process, employed in the spray-transfer mode, which bolsters the consistency of chemical composition while mitigating issues associated with distortion during welding [3,4,5]. A more gradual cooling pace within the critical transformation domain permits the hybrid laser-arc welding (HLAW) method to amalgamate a profound weld-penetration depth with a low power consumption innate to laser welding, thereby achieving heightened energy efficiency and metallurgical stability [6,7,8]. The harmonious interplay between the laser beam and the GMAW arc demonstrates the laser’s capacity to impact the ionization of the GMAW arc, consequently diminishing its resistance and amplifying the quantity of electron current carriers that contribute to the genesis of the arc plasma. Furthermore, it has been revealed that the energy absorption of the laser by the GMAW arc can be diminished, yielding a more profound penetration [9].

Nevertheless, as the variables in the hybrid welding procedure greatly surpass those of conventional arc welding or laser welding, the process development becomes increasingly intricate and costly [10]. Numerous experiments involving hybrid laser–GMA welding have been executed in an endeavor to ascertain the weldability of various base materials [11,12] and to examine the effects of operational parameters on the weld quality [13,14,15]. These experimental investigations have furnished valuable insights for comprehending and enhancing the hybrid welding technique. However, ascertaining the intricacies of the physics implicated in the observed phenomena proves unattainable through experimentation alone, given the opaque nature of metal, diminutive and unstable keyhole, elevated temperature plasma, and the potent coupling of welding parameters in a transient manner [6]. Concurrently, with the advancement of welding simulation technology, the computational-aided design methodology has been integrated into the development of novel welding processes [16,17,18]. Formation parameters, such as the width and depth of the weldment, are primarily taken into account when establishing the welding procedure [19]. Albeit the correlation between formation parameters and process parameters can be ascertained through a series of experiments, there exists an alternative cost-efficient approach, specifically, finite element thermal simulation [20,21,22]. Deng et al. [23] used the numerical simulation method to quantitatively compare the welding deformation of laser welding and gas shielded welding joints. The simulation results of the deformation mechanism are consistent with the experimental results, indicating that numerical simulation can effectively support the analysis of the laser–GMAW welding mechanism.

In the current study, the consequential impacts of the central welding parameters during the laser–GMAW hybrid welding procedure, on the formation characteristics and attributes of 8 mm AH36 T-joint welds, were minutely examined by a streamlined combination of experimental and simulation techniques. Commercial finite element ANSYS R15.0 software was used for numerical simulation in our research. Melding these experimental and simulation outcomes of welding thermal cycles, the influences of various process parameters, including laser power, welding velocity, wire feed rate, defocusing distance, and laser-wire distance, were scrutinized in depth. Therefore, this research is important for analyzing the influence of process parameters on welded joints, which could be useful for the subsequent design of laser–GMAW hybrid welding process parameters.

## 2. Methods of Simulation and Experiment

### 2.1. Simulation Model

#### 2.1.1. Thermal Conduction Model

Subordinately accounting for the mode of heat conduction, we employ the finite element methodology for simulating the thermal dynamics implicated in the hybrid laser–gas metal arc welding (GMAW) of T-joints embodied in 8 mm thick steel plates. The deciphering of the temperature field in this exposition leans upon the classical predilection for the Fourier heat equation oriented towards spatially three-dimensional transient heat conduction. Accordingly, the temperature calculations in this work adopt the classical Fourier’s heat equation for 3D transient heat conduction [24]. This equation is given as

(1) $$\frac{\partial }{\partial x}\left(\lambda \frac{\partial T}{\partial x}\right)+\frac{\partial }{\partial y}\left(\lambda \frac{\partial T}{\partial y}\right)+\frac{\partial }{\partial z}\left(\lambda \frac{\partial T}{\partial z}\right)=\rho \;C_{p}\frac{\partial T}{\partial t}-q$$

where *ρ* represents density, *C_p_* is specific heat capacity, *λ* is thermal conductivity, *t* represents welding time, *x*, *y,* and *z* represent space coordinates, *q* is the volumetric internal energy generation, and *T* is temperature.

#### 2.1.2. Heat Source Model

**Figure 1:**
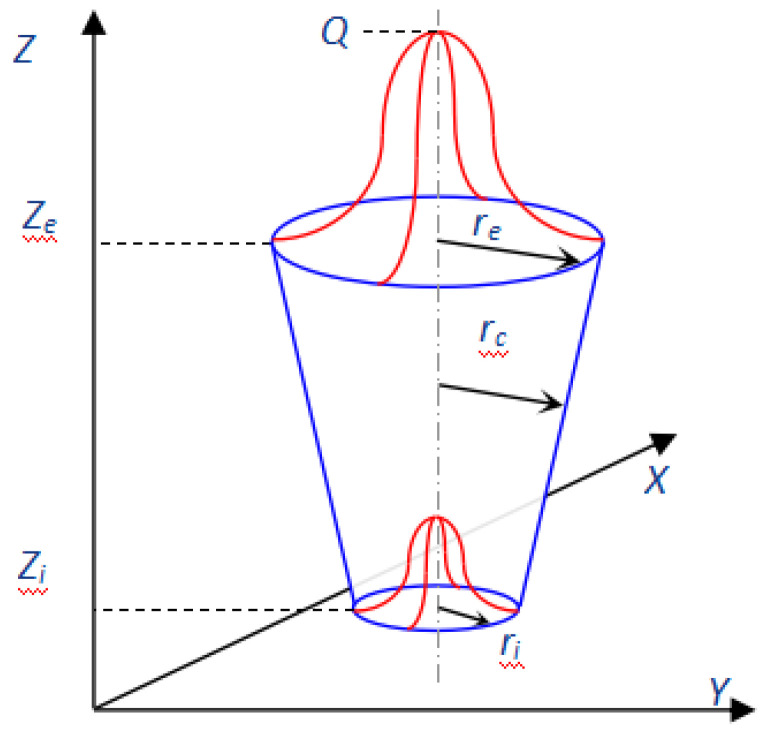
Sketch Map for Conical Heat Source.

In the course of the fusion process, the absorption of energy is not distributed homogeneously throughout the depth. Thermal energy diminishes progressively as the depth incrementally expands. The conical heat source model is a rotational energy source that exhibits a Gaussian distribution of thermal flux within the radial domain alongside a distinctive attenuation trajectory in the depth domain, thus reflecting the inherent attributes of actual laser fusion procedures, as depicted in Figure 1. The conical heat source model can be numerically articulated for a maneuvering laser trajectory in the ‘*x*’ direction, as indicated in the subsequent equations [24].

(2) $$q_{L}=\frac{9Qe^{3}}{\pi (e^{3}-1)(Z_{e}-Z_{i})(r_{e}^{2}+r_{e}r_{i}+r_{i}^{2})}\exp(-\frac{3\left[{\left(x-vt\right)}^{2}+y^{2}\right]}{r_{c}^{2}})$$

(3) Q=ηP

(4) $$r_{c}=f(z)=r_{i}+(r_{e}-r_{i})\frac{z-z_{i}}{z_{e}-z_{i}}$$

where *q_L_* is the laser heat flux, *z_e_*, *z_i_*, *r_e_* and *r_i_* represent the z-coordinates and radii of the top and bottom surfaces, respectively, *r_c_* is the distribution parameter, *e* is the base of the natural logarithm, *Q_L_* represents the effective laser power, and *η_L_* is the laser heat efficiency, 0.8 in this calculation.

**Figure 2:**
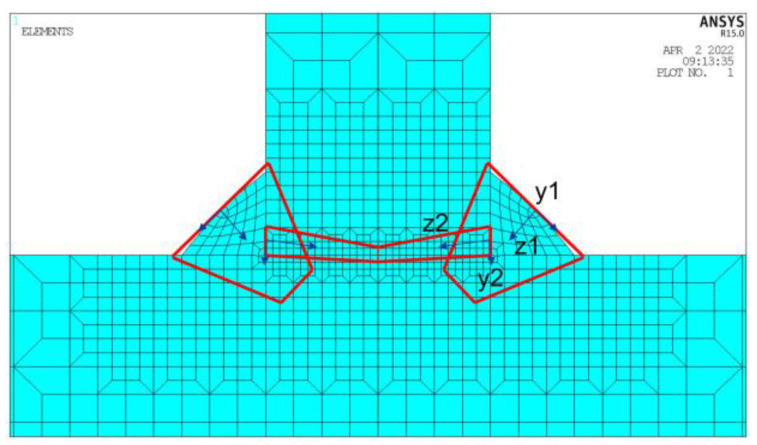
Laser–GMAW Hybrid Heat Model.

When employing a model of a conical heat source as a proxy for simulating arc and laser dynamics, it is critical to note the disparity in the coordinate systems of these two distinct heat sources. The procedure undertaken to simulate the thermal field during the welding process necessitates a discrete application of the arc and laser heat, achieved through the alteration of their respective coordinate systems. A graphical representation of this dual heat source model can be observed in Figure 2; the mathematical formulation is shown in Equation (5) as follows, where *r*_1_ and *r*_2_ represent the arc and laser heat distribution, respectively.

(5) $$q=\left\{\begin{array}{c} \frac{9Q_{A}e^{3}}{\pi (e^{3}-1)(Z_{f}-Z_{m1})(r_{f}^{2}+r_{f}r_{m1}+r_{m1}^{2})}\exp(-\frac{3\left[{\left(x-vt\right)}^{2}+y^{2}\right]}{r_{1}^{2}})\\ \frac{9Q_{L}e^{3}}{\pi (e^{3}-1)(Z_{m1}-Z_{m2})(r_{m1}^{2}+r_{m1}r_{m2}+r_{m2}^{2})}\exp(-\frac{3\left[{\left(x-vt\right)}^{2}+y^{2}\right]}{r_{2}^{2}}) \end{array}\right.$$

**Figure 3:**
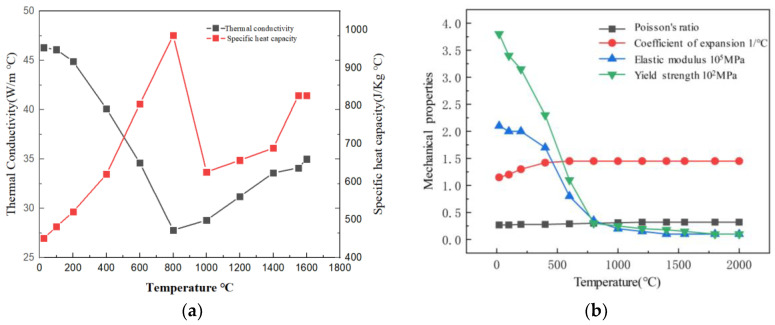
Thermal Physical Properties of AH36 Steel. (**a**) Incident Position P1 (Thermophysical properties). (**b**) Incident Position P2 (Mechanical properties).

Copious alterations were made, utilizing material physics simulation software Jmatpro V10, to ascertain the thermophysical attribute parameters of AH36. The steel model in question is AH36, with its thermal, physical, and mechanical performance indicators on display in Figure 3.

#### 2.1.3. Numerical Model

**Figure 4:**
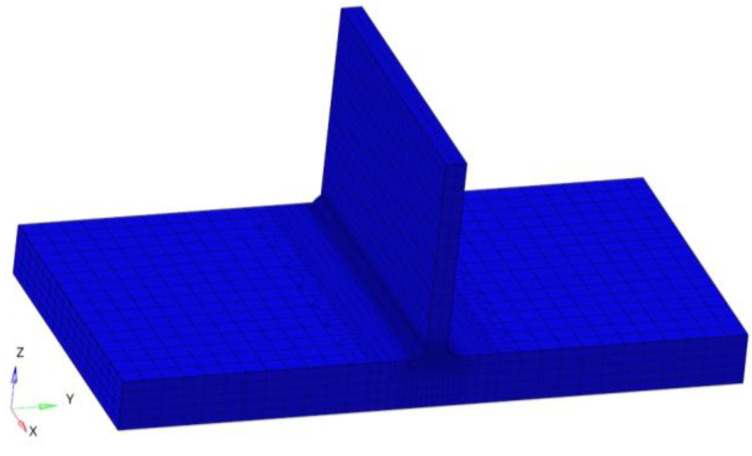
Finite Element Model for T-joints.

Three-dimensional solid constituents, identified as solid70, employing the octahedral integration technique for quadrature, are utilized for the numerical model construction. The process of welding inherently involves rapid temperature fluctuations concerning both spatial and temporal dimensions, particularly displaying pronounced temperature gradients. Consequently, non-uniform grids are employed for the discretization of the domain. The distribution encompasses the weld material as well as its immediate vicinity, with subdivisions incorporated for increased detail. Conversely, regions situated at a considerable distance from the weld seam exhibit a coarser grid composition. The partitioning scheme is depicted in Figure 4, comprising 54,912 elements and 59,930 nodes. To accurately compute welding-induced deformations and residual stress, the thermo-elastic-plastic finite element methodology is adopted. Furthermore, the resultant temperature field is utilized as the heat input parameter. The welding residual stress and concomitant deformation can be derived via the iterative coupling of thermal and mechanical calculations.

**Figure 5:**
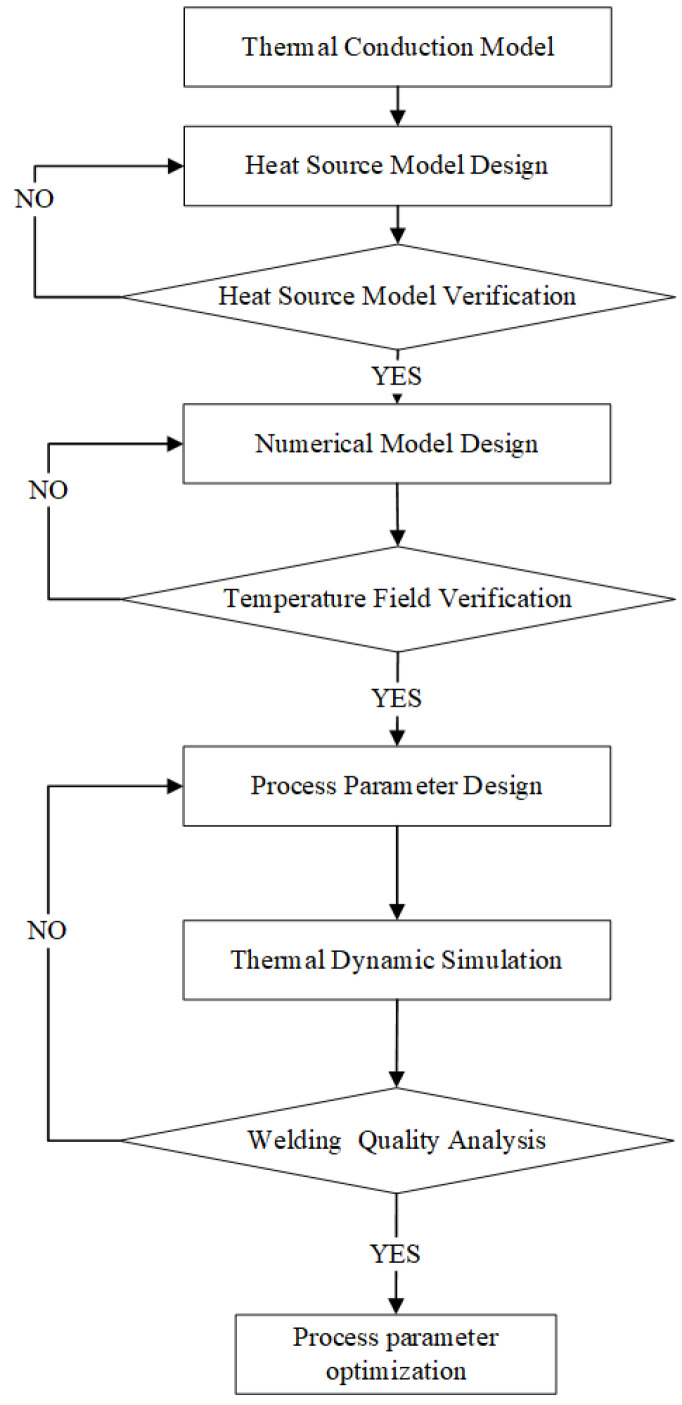
Numerical Simulation Methodology Flowchart.

The numerical simulation methodology flowchart is shown in Figure 5. Firstly, the composite welding heat source model is constructed using the classic conical heat source model. We optimize the parameters of the heat source model based on experiment data of the welding seam. Then, the numerical model is adjusted with the resolution and accuracy of the temperature distribution in the regions of severe thermal gradients. At last, we will design different process parameters to do the thermal dynamic simulation and optimize the process parameter by the analysis of welding quality.

### 2.2. Experimental Setup

#### 2.2.1. Experimental Parameters

The elemental constitution of the base metal (BM) and the conduit (ER70S-6) applied to the high-laser arc welding (HLAW) methodology are delineated in Table 1. Depicting the high-yield configuration of the laser apparatus for the fusion procedure is Figure 1. A diode-driven Yb: YAG disk laser (TRUMPF, TruDisk 10003 laser, Wuhan, China), operating on a persistent wavelength of 1030 nm, an impressive beam quality of 8/12 mm Ámrad, and a maximum capacity of 10 kW, was engaged for the laser welding operation. The laser cranium was armed with a focal length of 300 mm, bestowing a laser spot diameter of 0.6 mm at the point of convergence. The welding procedure was accomplished by placing the laser at the leading position, with the arc trailing. The specimens were purged with acetone before the welding procedure. The laser cranium was adjusted 5° from its perpendicular axis to safeguard it from the rear reflection of light. Argon was utilized as the protective gas to shield the welding zone, with a device specifically constructed to stay within approximately 5 mm of the laser spot at the leading position. The discharge rate of the protective gas was chosen to be 0.52 MPa.

#### 2.2.2. Experimental Design

We executed longitudinal frame laser fusion welding procedural experiments employing a laser composite welding apparatus. As slender plates of 8 mm are prevalently employed as thin plate structures, we carried out investigative studies on 8 mm longitudinal corner joints to confirm the influence of process parameters on the quality of the weld. Throughout the empirical process, single variable adjustments were made to the four core procedural parameters: the potency of the laser, the velocity of wire feeding (arc power is incapable of direct control within the experimental context, but it maintains a proportional relationship to wire feeding velocity, hence the adjustment variable employed is the feeding speed of the wire), speed of welding, and the incident height. The configurations for the procedural parameters on steel plates of 8 mm thickness are delineated in Table 2. With welding gun inclinations of 33.5 degrees, all other procedural parameters remain consistent, which include a defocus of +5 mm, laser-wire distance of 3 mm, wire extension of 18 mm, and a wire feeding angle of 40 degrees.

Simultaneously, a macroscopic metallographic examination is carried out on 8 mm T-joint laser–GMAW weld seams to scrutinize the impact of varying process parameters on the formation of said laser–GMAW weld seams.

## 3. Results and Discussions

### 3.1. Verification of Heat Source Model

**Figure 6:**
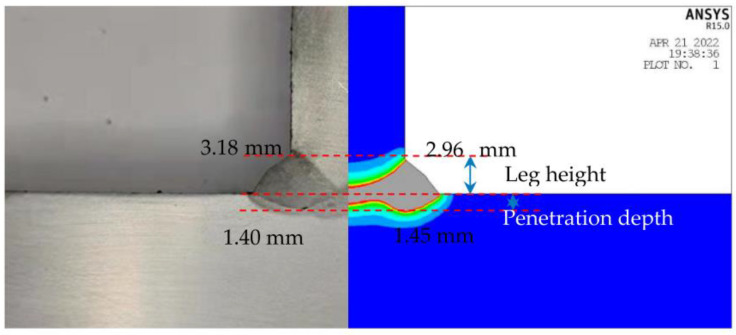
Seam Cross-Section Morphology Comparison between Simulation and Experiment Result.

Figure 6 illustrates a comparative analysis of the seam cross-sectional morphology between the simulated outcomes and experimental findings of 8 mm T-joint laser–GMAW welding. Upon scrutinizing the cross-sectional configurations, there is a discernable degree of correspondence between the two results, with a deviation of less than 8% amid the characteristic dimensions of leg height and penetration depth. This substantiates that the simulation is proficient in mirroring the experimental data accurately.

### 3.2. Effects of Incident Position

**Figure 7:**
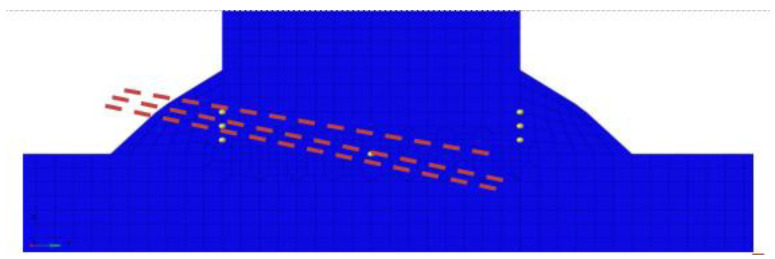
Cross-Section Grid Diagram.

Portrayed in Figure 7 is a transverse schematic of 8 mm T-joints. The inclination traced (dashed lines in the Figure 7) between the demarcated line and the horizontal trajectory within the depiction corresponds to the angle of laser incidence, a precise 12°. This particular angle of approach is dictated by the established WPS (Welding Procedure Specification). Noteworthy points P1, P2, and P3 as designated in Figure 6 signify the incidence positions located at varying distances of 0.5, 1, and 1.5 mm, respectively, from the base slab. Upon the convergence of the incidence placement and trajectory, it can be inferred that P2 is situated in closest proximity to the median point. The positional differences of points P1 and P3 are approximately 0.7 mm when measured from the central midpoint. In compliance with the procedural parameters as established by the WPS (refer to Table 3), the thermal domains following the application of laser–GMAW hybrid welding are simulated across the aforementioned three incidence points.

**Figure 8:**
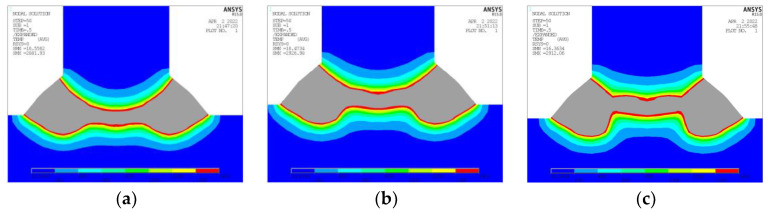
Temperature Nephogram at Different Incident Positions. (**a**) Incident Position P1. (**b**) Incident Position P2. (**c**) Incident Position P3.

The temperature nephogram depicted in Figure 8 illustrates the correlation between the alterations in the temperature field and the location of incidence. The dashed demarcation within the gray region (temperature > 1450 °C) signifies that the interface temperature between the longitudinal frame and the base plate fulfills the melting prerequisites. This allows the T-joints to establish a robust connection. If the dashed line resides exterior to the gray area, the interface will either not melt or experience partial melting, consequently giving rise to a flawed joint. When the angle of incidence is at 12°, both points of incidence, P1 and P2, can undergo successful fusion. The melting zone of the incident point P3 is situated above the dashed line, and the temperature at said line does not achieve the requisite melting temperature. As a result, an adjustment to the incident position is necessitated. In general, it is observable that with the ascension of the incident position height, the gray temperature area (temperature ≥ 1450 °C) progressively migrates towards the longitudinal flank. Should the height be excessively elevated, it easily incites the occurrence of intermediary insufficient penetration.

**Figure 9:**
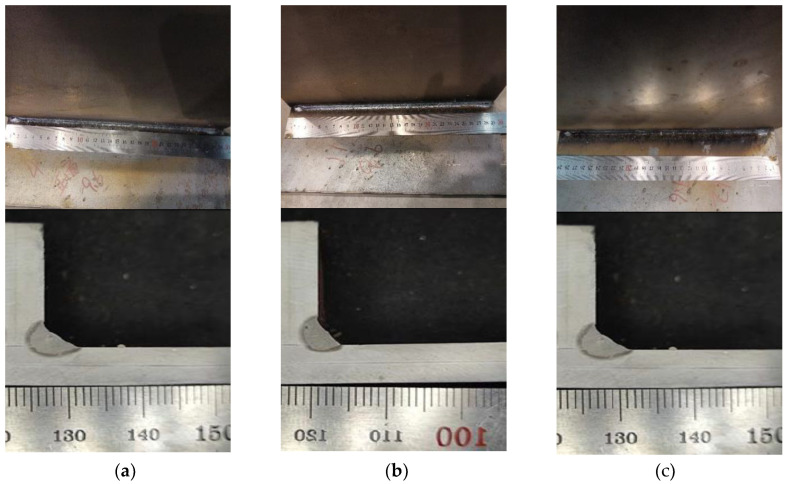
Experiment Result at Different Incident Positions. (**a**) Incident Position P1. (**b**) Incident Position P2. (**c**) Incident Position P3.

Illustrated in Figure 9 are the outcomes of experimental trials under varied points of incidence, with all other parameters held constant. These include a laser power of 5000 W, a wire feeding velocity of 11.8 mm per minute, and a welding speed of 1.5 m per minute. It may be inferred from the data that at the points of incidence of 0.5 mm (as depicted in Figure 9a) and 0 mm (Figure 9b), the formation of the welding seams proved satisfactory. Elevating the point of incidence to 1.5 mm (Figure 9c), however, contributes to the occurrence of sporadic weld beads. Consequently, an incident position of 0.5 mm is selected for the subsequent welding tests.

### 3.3. Effects of Laser Power

The laser serves as a primary heat source in hybrid welding, and the impact of its power on weld dimensions is of significant importance. Previous research has indicated that the surface and near-surface temperature fields are governed by the arc heat source, whereas the deep temperature regions are dictated by the laser heat source. As such, for T-joint welding, the most crucial region is the intersection of the longitudinal frame and bottom plate situated in the center. The ability of the temperature in this region to reach the melting point is not solely influenced by the position and angle of incidence but is also contingent upon the laser power.

**Figure 10:**
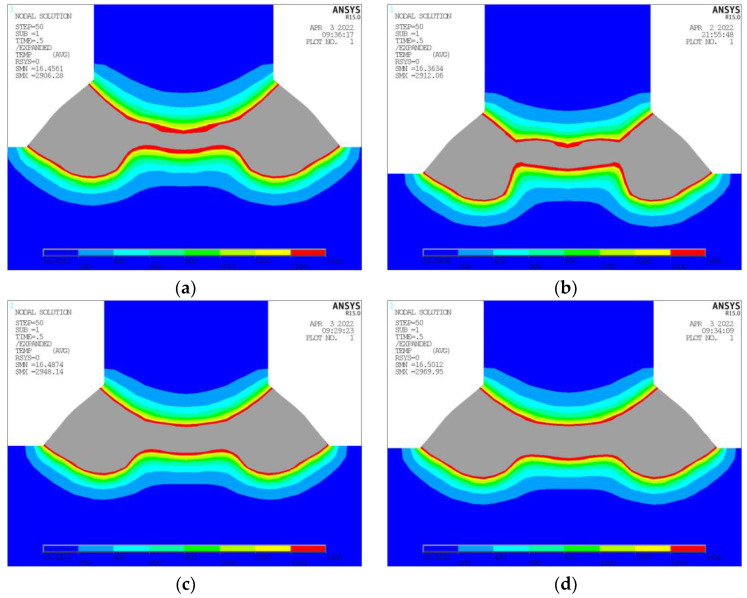
Temperature Nephogram at Different Laser Power. (**a**) Laser Power 4200 W. (**b**) Laser Power 4700 W. (**c**) Laser Power 5200 W. (**d**) Laser Power 5700 W.

Exemplified in Table 4 are the computational outcomes resulting from alterations in laser power alone, with other parameters remaining consistent, specifically, an arc power of 7000 W, welding speed of 2500 mm/min, incidence position of 1.5 mm, and incidence angle of 12°. Figure 10 depicts the cross-sectional temperature nephograms under varying laser power levels. It can be observed that when the laser power is 4200 W, the joint surface between the longitudinal frame and bottom plate has not entirely reached its melting temperature. Upon increasing the laser power to 4700 W, the joint surface attains the full melting temperature; however, the minimum melting depth is rather shallow, posing a risk for partial welding during the actual process.

**Figure 11:**
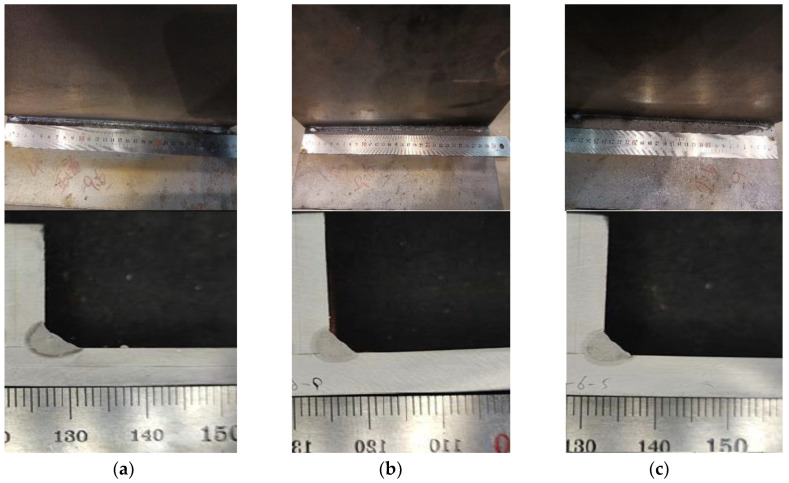
Experiment Result at Different Laser Power. (**a**) Laser Power 5000 W. (**b**) Laser Power 6000 W. (**c**) Laser Power 4000 W.

As the laser power is further elevated to 5200 W and 5700 W, the bottom plate region achieving the melting point temperature progressively expands, accompanied by a gradual increase in the minimum melting depth. Concurrently, the welding legs remain predominantly unaffected, with fluctuations primarily attributable to measurement errors. Figure 11 illustrates the experimental outcomes under disparate laser power levels, maintaining consistent additional parameters, which include a wire feeding speed of 11.8 mm/min, welding speed of 1.5 m/min, and incidence position of 0.5 mm. By the experimental findings, satisfactory welding seam formation occurs when the laser power amounts to both 5000 W (Figure 11a) and 6000 W (Figure 11b). Conversely, when the laser power is reduced to 4000 W (Figure 11c), there is a discernible narrowing of the welding seam.

**Figure 12:**
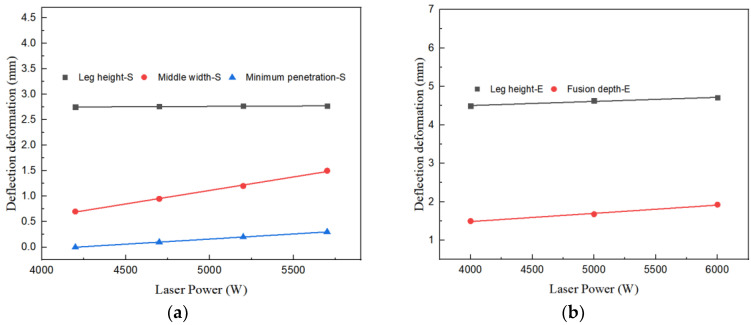
Relationship between Laser Power and Welding Seam Feature Size. (**a**) Simulation Fitting Curve. (**b**) Experiment Fitting Curve.

Moreover, an observation can be deduced that concomitant with the escalation of laser power, the characteristic dimension of both the experimental trials and the simulation corresponding to disparate laser powers showcased in Table 5 exhibit an elevation. Upon fitting the simulation specific parameters, the outcomes have been illustrated in Figure 12a. A discernible pattern is that all characteristic dimensions, bar welding leg, undergo an expansion with greater laser power, and this fluctuation approximates a linear relationship. The gradient of the line fitting the leg height stands at 1.48 × 10^−5^. The gradient corresponding to the middle width fitting line is evaluated as 5.32 × 10^−4^. This implies that for each 1000 W amplification in laser power, a subsequent increase in the middle width by 0.532 mm is seen. The gradient pertaining to the fitting line of minimal penetration is determined to be 2.38 × 10^−4^. It substantiates that for each supplemental 1000 W in laser power, the least melting depth experiences an upturn by 0.238 mm. Adjusting the experimental specific parameters, the findings have been delineated in Figure 12b. Both welding leg and fusion depth rise commensurate with higher laser power. The gradient of the leg height fitting line is computed as 1.07 × 10^−4^, while the gradient of the fusion depth fitting line is determined to be 2.14 × 10^−4^.

### 3.4. Effects of Arc Power

**Figure 13:**
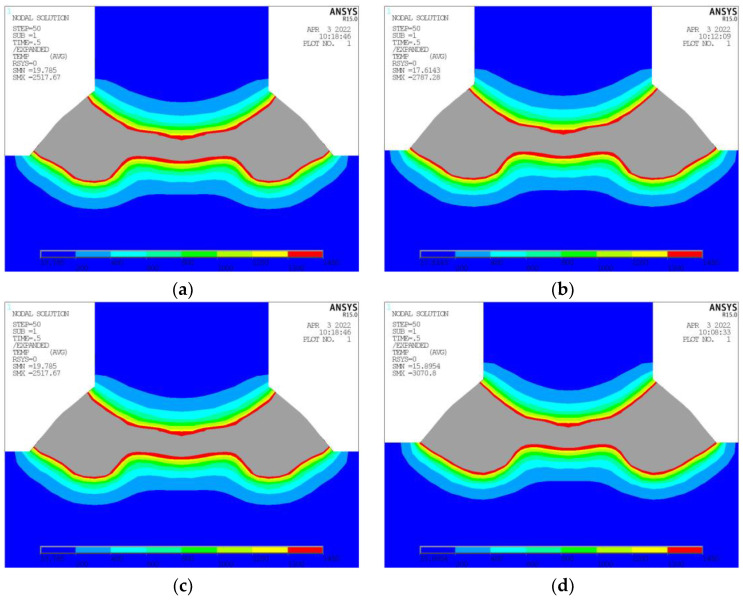
Temperature Nephogram at Different Arc Power. (**a**) Arc Power 5500 W. (**b**) Arc Power 6500 W. (**c**) Arc Power 7000 W. (**d**) Arc Power 7500 W.

**Figure 14:**
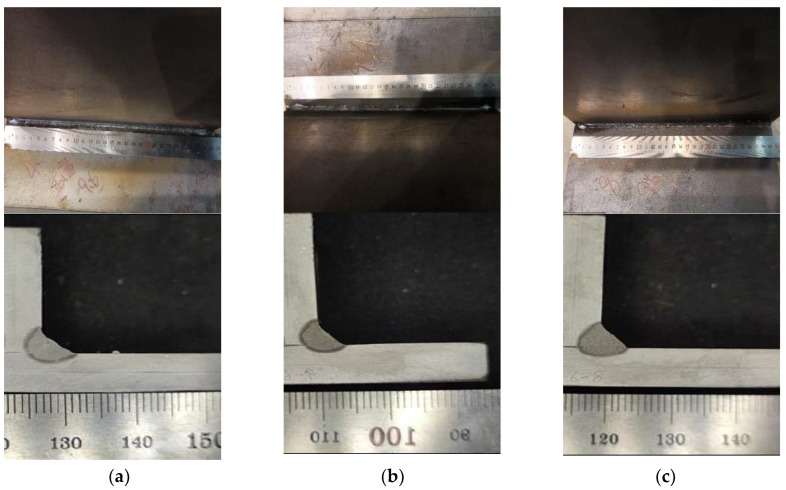
Experiment Result at Different Wire Feeding. (**a**) Wire Feeding 11.8 mm/min. (**b**) Wire Feeding 13.3 mm/min. (**c**) Wire Feeding 10.3 mm/min.

The electric arc constitutes a significant heat source during the process of hybrid welding. The role the arc’s power plays in determining the size of the weld is pivotal. In this particular simulation, the arc power alone varies, while other factors remain constant. These factors include the laser power, which stands at 4700 W, the welding speed of 2500 mm per minute, an incident position of 1.5 mm, and an incident angle of 12 degrees. An examination of the nephrogram shifts in Figure 13 reveals that fluctuations in arc power do not impact the central width or minimum penetration size. However, they do affect the overall size of the welding leg. The speed at which wire feeds can act as an indicator of arc power influence since a linear relationship exists between the two variables. As such, wire feeding speed was employed as a parameter to assess the arc power’s influence on laser–GMAW welding throughout the experiment. Figure 14a,d,e display the experimental results at varying wire feeding speeds while maintaining constant parameters such as the laser power of 5000 W, a welding speed of 1.5 m per minute, and an incident position of 0.5 mm. Upon reviewing the experimental results, a well-formed welding seam is evident at wire feeding speeds of 11.8 (Figure 14a), 13.3 (Figure 14b), and 10.3 mm per minute (Figure 14c).

**Figure 15:**
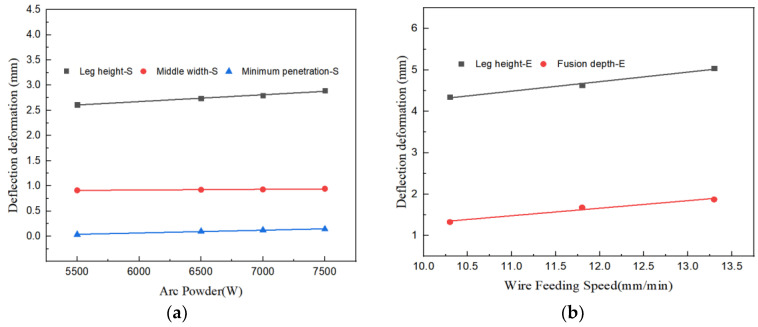
Relationship between Arc Power and Welding Seam Feature Size. (**a**) Simulation Fitting Curve. (**b**) Experiment Fitting Curve.

The correlative analysis depicted in Figure 15a elucidates a linear association between the arc power and the dimensions of the welding leg. The gradient of the fitted line amounts to 1.35 × 10^−4^, signifying that a 1000 W enhancement in arc power corresponds to a 0.135 mm augmentation in the welding leg’s magnitude. The impact of arc power on the minimal penetration depth and the median breadth remains comparatively negligible, with slopes of the fitted lines being 5.48 × 10^−5^ and 1.46 × 10^−5^, respectively. Upon assimilating the experimental characteristic parameters, the outcomes are exhibited in Figure 15b. As the wire feed velocity escalates, an upward trend is observed in both the welding leg and the fusion depth. The gradients of the respective fitted lines for leg height and fusion depth are 0.23 and 0.18.

### 3.5. Effects of Welding Speed

**Figure 16:**
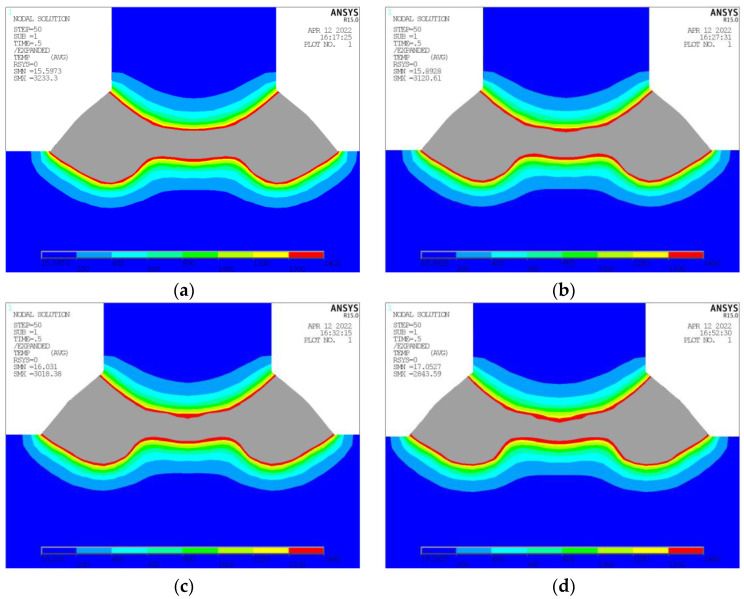
Temperature Nephogram at Different Welding Speed. (**a**) Welding Speed 2200 mm/min. (**b**) Welding Speed 2300 mm/min. (**c**) Welding Speed 2400 mm/min. (**d**) Welding Speed 2600 mm/min.

**Figure 17:**
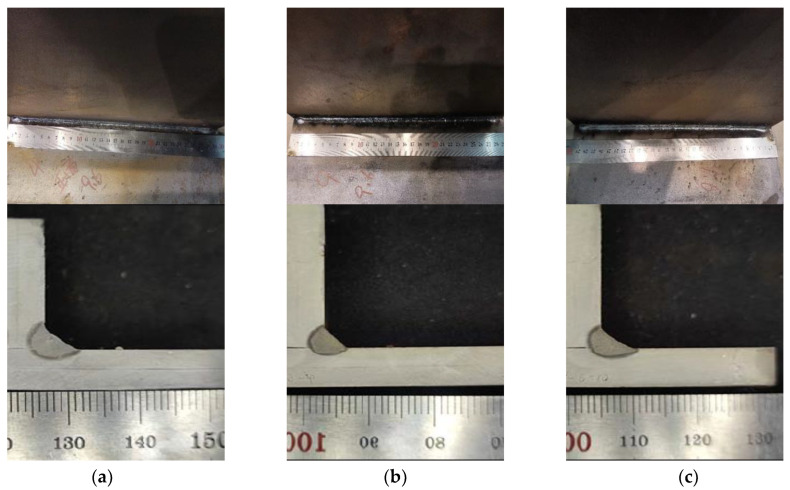
Experiment Result at Different Welding Speeds. (**a**) Welding Speed 1500 mm/min. (**b**) Welding Speed 1800 mm/min. (**c**) Welding Speed 1200 mm/min.

The outcome of the simulation, in which the welding speed is increased, is depicted in Figure 16. All other parameters remain constant; specifically, the laser power is set at 4700 W, arc power at 7000 W, incident position at 1.5 mm, and incident angle at 12°. As the welding speed escalates, the dimensions of the welding leg, including its height, middle width, and minimum penetration depth, consistently decline. Figure 17 exhibits the experimental findings at various welding velocities, with all other parameters kept consistent, such as a laser power of 5000 W, wire feeding rate of 11.8 mm/min, and an incident position of 0.5 mm. By the experimental evidence, the weld formation is well-executed across different welding speeds.

**Figure 18:**
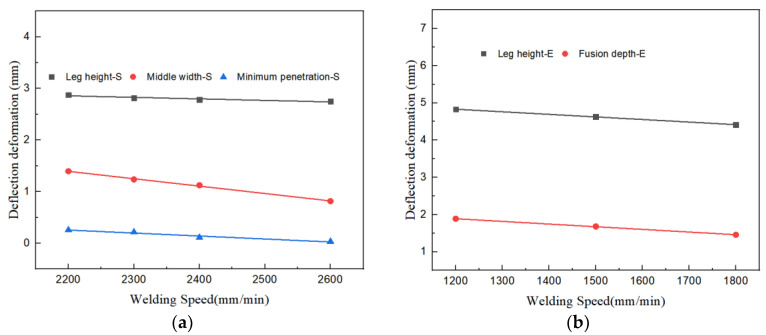
Relationship between Welding Speed and Seam Feature Size. (**a**) Simulation Fitting Curve. (**b**) Experiment Fitting Curve.

By correlating the simulation and experimental characteristic parameters in Table 6, the conclusions are presented in Figure 18. It is discernible that all characteristic parameters diminish as the welding velocity increases, and their fluctuation pattern is essentially linear. The slope of the fitting line for the welding leg height is −2.94 × 10^−4^, signifying that for every 100 mm/min reduction in welding speed, the height of the welding leg augments by 0.029 mm. The slope of the middle width fitting line is −1.43 × 10^−3^, indicating that for every 100 mm/min reduction in welding speed, the height of the welding leg elevates by 0.143 mm. The slope of the fitted line for the minimum penetration is −5.77×10^−4^, meaning that for every 100 mm/min reduction in welding speed, the height of the minimum penetration amplifies by 0.057 mm. By fitting the experimental characteristic parameters, the outcomes are demonstrated in Figure 18b. Both the welding leg and fusion depth diminish as the welding speed rises. The slope of the leg height fitting line is −6.91 × 10^−4^, and the slope of the fusion depth fitting line is −7.15 × 10^−4^.

### 3.6. Deformation Simulation Calculation

**Figure 19:**
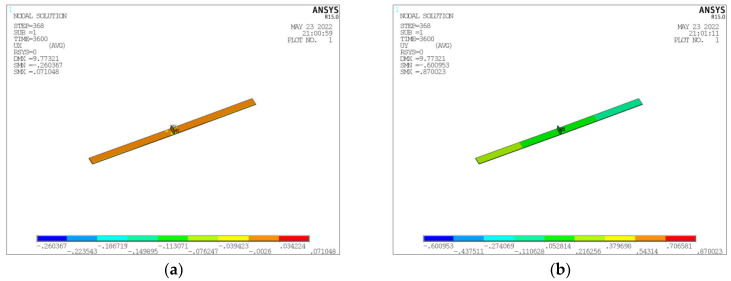

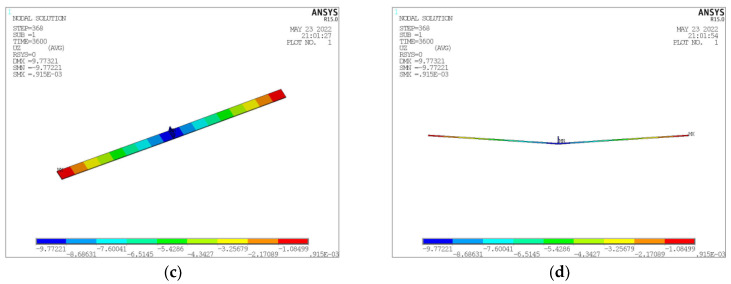
Welding Deformation Simulation Result in Free State. (**a**) Deformation in X Direction (**b**) Deformation in Y Direction. (**c**) Deformation in Z Direction. (**d**) Deformation in Z Direction Magnified by 10 Times.

**Figure 20:**
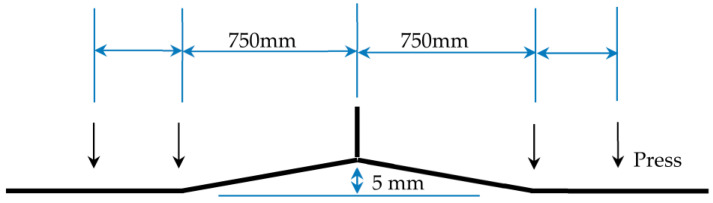
Reverse Deformation Simulation Settings for 8 mm T-joint Welding.

**Figure 21:**
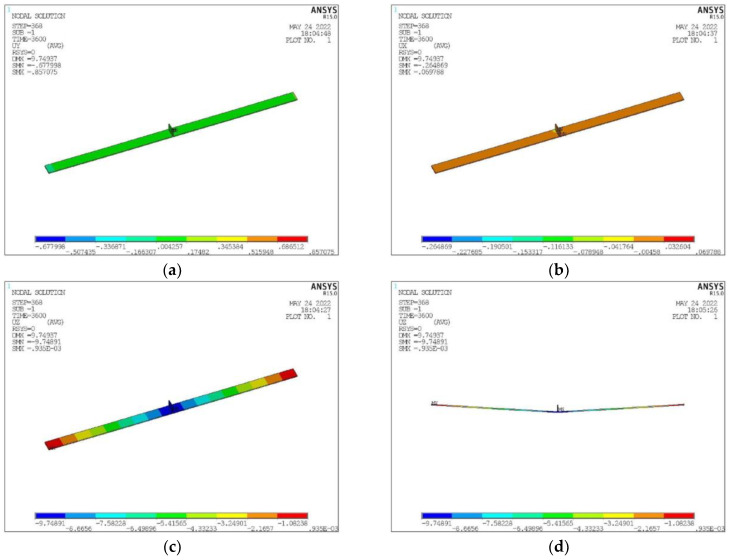
Simulation Results of Welding Deformation after Reverse Deformation Limitation. (**a**) Deformation in X Direction. (**b**) Deformation in Y Direction. (**c**) Deformation in Z Direction. (**d**) Deformation in Z Direction Magnified by 10 Times.

The determinants for formulating the deformation of 8 mm T-joint welding comprise an arc power of 7000 Watts, a laser power of 4700 Watts, and a welding velocity of 2500 mm per minute. In the unencumbered state of welding, the induced deformation is illustrated in Figure 19. Contraction deformation is primarily observed in the longitudinal and width dimensions, whereas comprehensive deformation is chiefly characterized by angular deformation in the Z direction. Distortion reaching a peak of 6.7 mm in the Z direction surpasses the established deformation threshold. Pursuant to the counteractive deformation steps exhibited in Figure 20, displacement limitation is imposed at a distance spanning 750–1170 mm from the center of the longitudinal framework. Furthermore, counteractive deformation is applied within the 0–750 range, incorporating a measure of 5 mm and a trajectory towards the Z-axis positive direction (as depicted in Figure 21a). Owing to the pre-set reverse deformation quantity, the deformation figure necessitates an increment of 5 mm. Figure 21b exemplifies that when the welding has cooled to ambient temperature, without the removal of the exterior load, the deformation value stands at −1.9 mm. This signifies that the deformation relative to the initial plane is 3.1 mm, featuring an arched center. Figure 21 showcases the release of the elastic strain upon the removal of the external load, with the maximum angular deformation being −6.4 mm. Subtracting the counteractive deformation of 5 mm results in a deformation rate relative to the initial plane that amounts to −1.4 mm, exhibiting a concave center that fulfills the designated requirements.

Observing from the standpoint of deformation control, in order to maintain the deformation of a 3000 mm wide and 8 mm thick ship longitudinal below 5 mm, it is imperative to introduce a 7 mm reverse deformation at the center. Additionally, displacement restrictions at positions ± 750 from the center are deemed necessary. Consequently, the ultimate deformation would amount to less than −2.1 mm angular deformation. After numerical simulations and experimental analyses, the optimal parameter range is shown in Table 7.

## 4. Conclusions

This discourse explores the impact of key attributes on the attributes of 8 mm T-joint laser–GMAW welds. Through simulation, the modulation of the thermal landscape and lengthwise deformation was scrutinized. The quality and salient proportions of the welding seam were analyzed empirically. Pondering upon these investigations, several deductions were made:

1. The thermodynamics of the 8 mm T-joint laser–GMAW welding were virtually recreated employing the twin-pyramidal heat source model. Subsequently, the resultant empirical welding seam architecture was juxtaposed with the simulated cross-section. The close congruity reinforced the veracity of the heat source model in question.
2. In the context of T-joint laser–GMAW welding, the incident angle and altitude are vital for determining the fidelity of the welding. As the incident angle escalates, the entire melt pool is displaced towards the base-plate lateral, thereby enhancing the molten expanse of the base plate. An excessive angle could pose a threat of partial welding on the flipside. An unduly high incident altitude would be susceptible to inadequate penetration of the interstitial joint surface.
3. The force of the arc and the feeding of the wire subtly influence the proportionality of the welding leg in the context of laser–GMAW welding; however, their perturbation over the deepest penetration and mid-width is diminutive. As the laser strength surges, the welding leg parameters remain irrevocably stable while the middle breadth and halcyon penetration register growth. Rising welding speed is observed to constrict the dimensions of the welding leg, middle-width, and minimal penetration recess.
4. The implementation of arched deformation mitigation in the middle of the T-joint laser–GMAW welding and confining the repositioning of the base plate at the optimal positions bilaterally can significantly curtail the welding deformation while bolstering the welding quality.

Through appropriate tweaking of the process parameters, the thermal consistency across the workpiece during the T-joint laser–GMAW welding process can be ameliorated, potentially reducing the welding deformation and amplifying the welding quality. However, numerical simulation cannot take all factors of a real environment into account, which cannot replace the welding procedure specification (WPS). Future inquiries can further fine-tune the process parameters through virtual modeling and tangible experiments, thereby catalyzing the welding process qualification.

## Figures and Tables

**Table 1:** Chemical Composition of Base Metal and Welding Wire [25].

Chemical Composition	C	Mn	Si	S	P	Nb	Cu
AH36	0.15~0.18	1.20~1.45	0.15~0.50	0.015	0.025	0.015~0.025	/
ER70S-6	0.06~0.15	1.40~1.85	0.80~1.15	≤0.035	0.025	/	<0.5

**Table 2:** AH36 Steel of 8 mm Welding Process Experiment.

No.	Laser Power (W)	Wire Feeding Speed (mm/min)	Welding Speed (m/min)	Incident Position (mm)
1	5000	11.8	1.5	0.5
2	6000	11.8	1.5	0.5
3	4000	11.8	1.5	0.5
4	5000	13.3	1.5	0.5
5	5000	10.3	1.5	0.5
6	5000	11.8	1.8	0.5
7	5000	11.8	1.2	0.5
8	5000	11.8	1.5	0
9	5000	11.8	1.5	1.5

**Table 3:** Process Parameters Corresponding to Different Incident Positions.

No.	Type	Laser Power (W)	Arc Power (W)	Wire Feeding Speed (mm/min)	Welding Speed (mm/min)	Incident Position (mm)	Incident Angle (°)
1	Simulation	4700	7000	/	2500	0.5	12
2	Simulation	4700	7000	/	2500	1	12
3	Simulation	4700	7000	/	2500	1.5	12
4	Experiment	5000	/	11.8	1500	0.5	12
5	Experiment	5000	/	11.8	1500	0	12
6	Experiment	5000	/	11.8	1500	1.5	12

**Table 4:** Feature Size Corresponding to Different Laser Power.

No.	Type	Laser Power (W)	Leg Height (mm)	Middle Width (mm)	Minimum Penetration (mm)	Fusion Depth (mm)
1	Simulation	4200	2.752	0.721	0.021	/
2	Simulation	4700	2.761	0.951	0.112	/
3	Simulation	5200	2.768	1.212	0.212	/
4	Simulation	5700	2.772	1.523	0.322	/
5	Experiment	5000	4.628	/	/	1.678
6	Experiment	6000	4.708	/	/	1.926
7	Experiment	4000	4.494	/	/	1.498

**Table 5:** Feature Size Corresponding to Different Arc Power or Wire Feeding.

No.	Type	Arc Power (W)	Wire Feeding Speed (mm/min)	Leg Height (mm)	Middle Width (mm)	Minimum Penetration (mm)	Fusion Depth (mm)
1	Simulation	5500	/	2.612	0.912	0.035	/
2	Simulation	6500	/	2.738	0.924	0.098	/
3	Simulation	7000	/	2.795	0.929	0.124	/
4	Simulation	7500	/	2.891	0.943	0.143	/
5	Experiment	/	11.8	4.628	/	/	1.678
6	Experiment	/	13.3	5.038	/	/	1.872
7	Experiment	/	10.3	4.349	/	/	1.327

**Table 6:** Feature Size Corresponding to Different Welding Speed.

No.	Type	Welding Speed (mm/min)	Leg Height (mm)	Middle Width (mm)	Minimum Penetration (mm)	Fusion Depth (mm)
1	Simulation	2200	2.876	1.397	0.257	/
2	Simulation	2300	2.814	1.236	0.216	/
3	Simulation	2400	2.783	1.125	0.112	/
4	Simulation	2600	2.751	0.816	0.035	/
5	Experiment	1500	4.628	/	/	1.678
6	Experiment	1800	4.414	/	/	1.457
7	Experiment	1200	4.829	/	/	1.886

**Table 7:** Optimal parameter range of 8 mm AH36 Steel welding process experiment.

Laser Power (W)	Wire Feeding Speed (mm/min)	Welding Speed (m/min)	Incident Position (mm)
5000–6000	10.3–13.3	1.2–1.8	0–0.5

## Data Availability

The data presented in this study are available on request from the corresponding author.
